# Uniqueness of gait kinematics in a cohort study

**DOI:** 10.1038/s41598-021-94815-z

**Published:** 2021-07-27

**Authors:** Gunwoo Park, Kyoung Min Lee, Seungbum Koo

**Affiliations:** 1 Department of Mechanical Engineering, Korea Advanced Institute of Science and Technology, 291 Daehak-ro, Yuseong-gu, Daejeon, 34141 Republic of Korea; 2 Department of Orthopedic Surgery, Seoul National University Bundang Hospital, Seongnam, Republic of Korea

**Keywords:** Mechanical engineering, Skeleton

## Abstract

Gait, the style of human walking, has been studied as a behavioral characteristic of an individual. Several studies have utilized gait to identify individuals with the aid of machine learning and computer vision techniques. However, there is a lack of studies on the nature of gait, such as the identification power or the uniqueness. This study aims to quantify the uniqueness of gait in a cohort. Three-dimensional full-body joint kinematics were obtained during normal walking trials from 488 subjects using a motion capture system. The joint angles of the gait cycle were converted into gait vectors. Four gait vectors were obtained from each subject, and all the gait vectors were pooled together. Two gait vectors were randomly selected from the pool and tested if they could be accurately classified if they were from the same person or not. The gait from the cohort was classified with an accuracy of 99.71% using the support vector machine with a radial basis function kernel as a classifier. Gait of a person is as unique as his/her facial motion and finger impedance, but not as unique as fingerprints.

## Introduction

Walking is a fundamental human movement that is personalized due to daily repetitive activities. Gait, a person’s walking pattern, is influenced by various individual features. For example, sex, somatotype, age, hand preference, habits, pathology, and ethnic or cultural background affect gait styles^1^. Physiological and behavioral characteristics, including gait, constitute the biometrics of a person. Biometrics are utilized in electronic banking and access control systems for identification and authentication. In security and forensic applications, the biometrics of gait have an advantage because they are non-invasive and easy to capture using cameras^2,3^.

One challenge in using biometrics of gait is that obtaining gait features such as the joint positions via video images is computationally expensive^3,4^. However, image processing and machine learning techniques have rapidly advanced in the last few years. Recent studies using deep neural networks and convolutional layers have extracted high-level gait features from images. Gait features are acquired in two main forms of data from a sequence of images: binary silhouette images and model-based kinematics of gait^5^. Binary silhouette images are usually processed into a gait energy image (GEI), which is the temporal accumulation of the silhouette sequence. A GEI can represent spatiotemporal information of a cyclic movement of a person, but the images should be captured from the side of the pedestrian^6,7^. Meanwhile, model-based gait kinematics estimation methods project and register a skeleton model to a person in the image using computer vision technologies, such as pose estimation^8–12^. By using a known camera angle or predicting the camera angle for the image, the three-dimensional posture of the skeleton model can be estimated to calculate gait kinematics^13–16^.

There is still a lack of research on the utility of gait as a biometric for identification. The performance of biometric systems is measured by evaluating how well the population set could be individualized. The top matches in the identification system and error rates of the verification system are evaluated for a selected dataset^17^. Unique characteristics are highly individualized but not vice versa. General uniqueness depends on the population size, number of features, and the difference between individual features^18^. Therefore, uniqueness cannot be assessed directly but can be estimated statistically^18–20^.

Biometric features should possess universality, distinctiveness, permanence, and collectability^4^. Popular biometric features such as fingerprint and face are accepted to have those properties. Moreover, new biometric features are being studied for identifying individuals. Benedikt et al.^21^ studied the uniqueness and permanence of facial movements acquired from three-dimensional video sequences. Armstrong et al.^22^ also investigated the uniqueness, permanence, and collectability of event-related potentials using electroencephalogram. Noh et al.^23^ presented ratiometric finger impedance as an alternative biometric feature. Multimodal biometric systems combine various biometric features to overcome the performance limitations of single-modal methods^4,24^. Development of multi-modal biometrics increased the importance of suggesting and validating other unique features from individuals.

Gait is not a particularly distinctive biometric feature but can be applied to systems with lower security requirements^4^. Nevertheless, discussion regarding the uniqueness of gait and its potential for individualization has been lacking. Many studies have directly assessed GEIs. Shiraga et al.^25^ proposed a convolutional neural network for view-invariant gait recognition. Zhang et al.^26^ introduced metric learning with Siamese neural networks, which were originally designed for few-shot image classification. For the model-based approach, studies for reconstructing three-dimensional kinematic models^8–12^ and using the kinematic models for person identification^13–16^ were developed. Liao et al.^14,15^ proposed a two-stage gait recognition method that first reconstructs 2D or 3D poses from images and then extracts features based on the sequence of poses. Instead of using a stick figure model, Li et al.^16^ adapted a vertex-based model to use body shape and pose during walking to identify individuals. These studies concluded that gait identification can be achieved by extracting the discriminative features of gait. However, it is necessary to further study the uniqueness of gait features and to analyze the benefits and limitations of using gait features for biometric identification.

Although vision-based 3D human pose estimation has been widely investigated, the standard method to obtain accurate human poses is to use an optical motion capture system with multiple synchronized cameras. The cameras can record the trajectories of the reflective skin markers attached to a subject. The motion capture system can provide a sub-millimeter accuracy in tracking the 3D position of a marker if an appropriate number of cameras and camera resolution are provided for a target volume^27,28^. The joint angles of the subject can be estimated by fitting the markers to a human model using the inverse kinematics method. It is significantly used in sports biomechanics^28^ and clinical biomechanics^29^ to quantify human kinematics.

In this study, we aim to explain the variation of gait data over a cohort of 488 subjects and estimate their uniqueness using gait kinematics data obtained using a motion capture system. There have been many attempts to use the gait features captured through videos for security and forensics. This study provides a statistical basis and analysis for utilizing gait features in applications such as biometric security systems and forensics through following steps:

- Extraction and interpretation of kinematic features of gait from the statistical distribution of cohort data using principal component analysis (PCA).
- Comparison of inter-subject variance and intra-subject similarity to check discriminative gait kinematics.
- Examination of binary classification methods to distinguish whether a given pair of gait kinematics is from the same person or from different persons.

## Methods

### Gait kinematics acquisition

This study was approved by the institutional review board at Seoul National University Bundang Hospital. Five hundred healthy subjects were recruited from the city of Seongnam, South Korea. Informed consent was obtained from all participants. Subject data acquisition and processing were performed in accordance with the institutional ethical guidelines of human subject research. Three-dimensional gait kinematics were recorded from the participants using a motion analysis system (Motion Analysis Corporation, Santa Rosa, California, USA) equipped with 10 cameras at 120 frames per second and two force plates. A sole operator having 9 years of experience placed photo-reflective skin markers in accordance with the Helen Hayes marker set. The study subjects walked barefoot on a 10-m long track at a self-selected comfortable speed after 10 walking trials. Other spatiotemporal gait parameters were also collected, including cadence, step length, and walking velocity.

Among the motion capture data from 500 subjects, the data from 12 subjects had technical problems in extracting three-dimensional gait kinematics due to missing data values or marker mismatches. Thus, data from 488 subjects were used for the study. Two-hundred forty-eight of them were male [average 36.8 (SD 17.2) years old, average 172.1 (SD 6.6) cm in height, and average 71.3 (SD 11.7) kg in weight] and 240 of them were female [average 37.4 (SD 16.3) years old, average 159.4 (SD 5.7) cm in height, and average 56.8 (SD 9.4) kg in weight].

**Figure 1:**
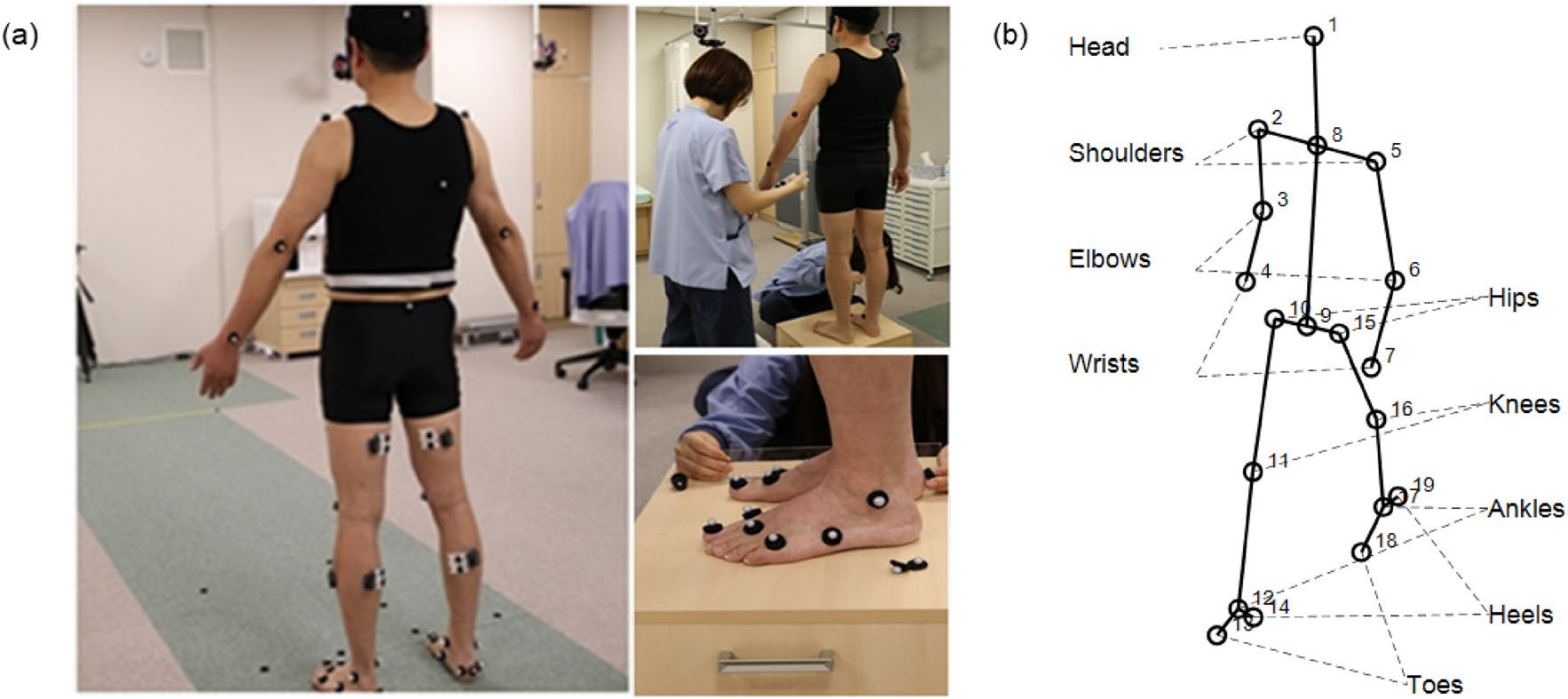
**(a)** Using Helen Hayes Marker set to capture the motion during walking trials. **(b)** Seventeen anatomical points were extracted from the motion capture data. Sixteen body segments (solid line) were defined by connecting the anatomical points.

Two walking trials were recorded for each subject. As shown in Fig. 1, we extracted the temporal locations of seven anatomical points in the upper body (head, shoulders, elbows, wrists) and 10 anatomical points in the lower body (hips, knees, ankles, toes, and heels) using the motion capture data. A body segment was defined between the two adjacent anatomical points. The temporary mid-hip and mid-shoulder points were determined using the left and right hip and shoulder points, respectively, to calculate the neck and trunk segments. Therefore, 17 points and 16 segments were extracted for each frame of the motion capture data. From the two walking trials, four gait cycles from the right heel strike to the next right heel strike were extracted.

### Gait kinematics data pre-processing

On average, a gait cycle took 1.03 s with 123.16 frames. For temporal normalization, we resampled each gait cycle using MATLAB (Version 2019a, MathWorks, Natick, Massachusetts, USA) linear interpolation to obtain 101 frames. The spatial coordinates of the 17 points were aligned to the laboratory coordinate system so that the positive X-axis was the direction of progression and the positive Z-axis was aligned vertically upwards.

**Figure 2:**
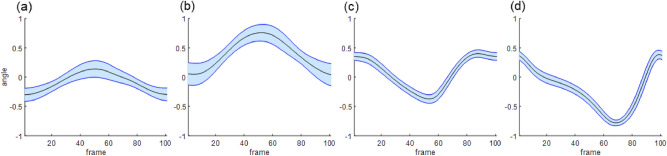
x-directional cosine plot of the segments. From left to right, each segment corresponds to the right upper arm **(a)**, right lower arm **(b)**, right upper leg **(c)**, and right lower leg **(d)**.

Accordingly, the orientations of each body segment were represented as direction cosines in the laboratory coordinate system, as shown in Fig. 2. The orientations of the segments in a cycle were transformed into a gait vector with 4848 components (16 segments × 3 dimensions × 101 frames). The gait vectors from 1952 gait data (488 subjects × 4 gait data) were collected.

The dimensions of a gait vector, 4848 dimensions, were reduced using the PCA method to create a low-dimensional gait feature vector. The 1952 gait vectors were created in the subspace of the original data space with 4848 dimensions. The subspace was spanned using 1951 principal components. The output of the dimensionality reduction was the loading matrix, score matrix, and mean gait vector. The loading matrix contained 1951 principal components in the order of variance. The score matrix had scores for the 1951 principal components to reconstruct the gait vector. We varied the number of principal components to understand the effectiveness of the dimensionality reduction. The dimension of the gait vector was reduced to three different levels that could explain 90, 95, and 99% of the total variance of the data, respectively.

### Verification models with binary classification

Two gait feature vectors from the 1952 gait feature vectors were compared to calculate their variance or similarities. We assumed that the magnitude of variance was smaller if a pair of gait feature vectors were from the same person. In our research, we set a verification problem as a binary classification task to determine whether the pair is from the same person or not. If the variance in the two gait feature vectors was within the classification boundary, they were considered to be from the same person.

From 488 subjects with four trials each, we obtained 2928 (488 × Comb(4, 2)) cases for the pairs of gait feature vectors from the same person and 1,901,248 (Comb(488, 2) × 4^2^) cases for the pairs of gait feature vectors from different persons. We subsampled 2928 cases among other cases. In summary, a total of 5856 cases were used for analysis. We tested the L1 and L2 norms, which are known as the Manhattan distance and Euclidean distance, respectively, to calculate the difference between the two gait feature vectors. Further, we tested the SVM for a more flexible boundary classification.

After learning the boundaries, we calculated the receiver operating characteristics (ROC) to validate the performance of the classifiers and observe their behaviors for varying boundaries. The false acceptance rate (FAR), false rejection rate (FRR), and equal error rate (EER) were calculated from the ROC. The FAR is the conditional probability that a pair of data was predicted to be from the same person while it was from two different persons. The FRR is the conditional probability that a pair of data was predicted to be from two different persons, while it was from the same person. The EER, a popular performance measure of biometrics research, is defined as the probability that the FAR and FRR are equal^17^.

We tested five classification methods using MATLAB. L1 norm, L2 norm, and SVM with linear and polynomial kernels were tested with gait feature vectors whose components were the absolute values of the original gait feature vectors. An SVM with a radial basis function (RBF) kernel was tested on the original gait feature vectors. We did not apply any additional normalization to the gait feature data because the mean of the gait feature vectors was zero. We used fivefold cross-validation for each classifier because there were no hyperparameters to be optimized.

## Results

### Interpretation of principal components

**Figure 3:**
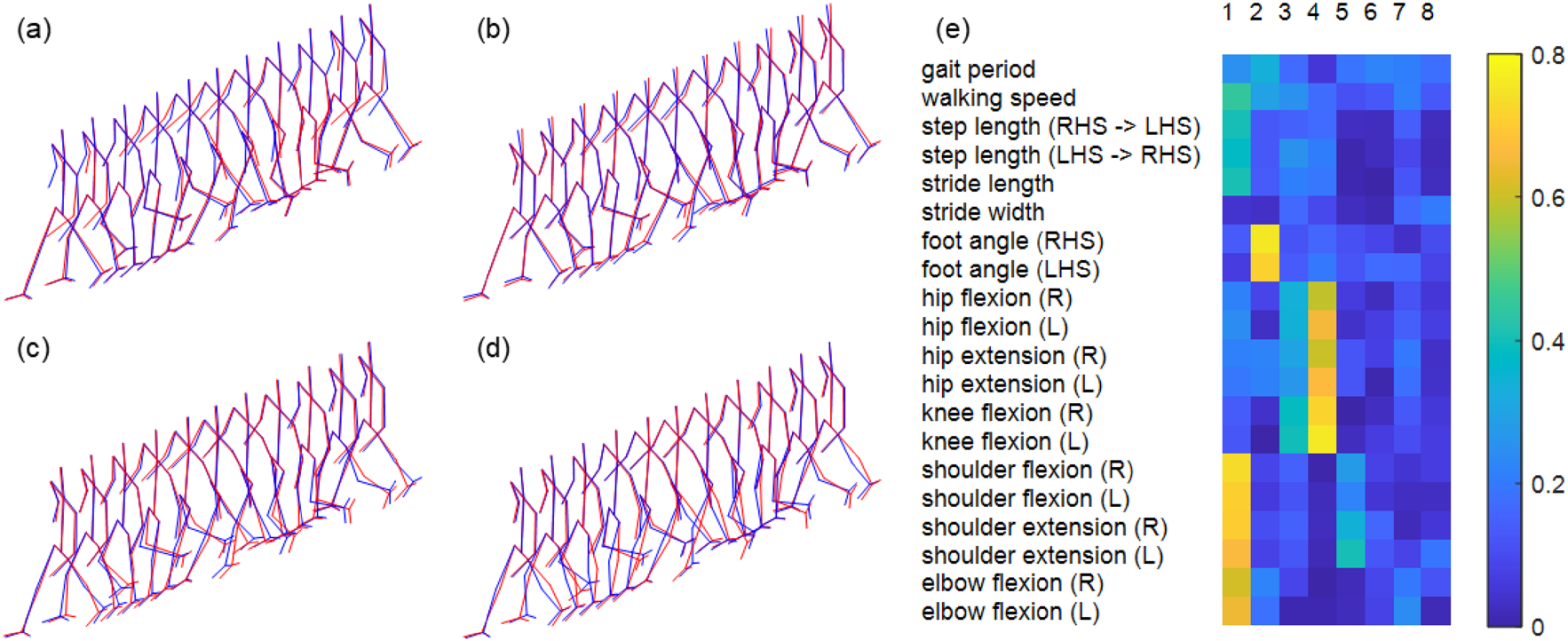
**(a–d)** Effect of the first four principal components were visualized by motion varying scores corresponding to each principal component. Each figure is about the first **(a)**, second **(b)**, third **(c)**, and fourth **(d)** principal component. The motion represented with red and the blue lines indicate the gait reconstructed by adding and subtracting the principal component from the mean gait. **(e)** Correlation of the principal components and clinical gait parameters was shown as a colormap.

We extracted 1951 principal components from 1952 cycles of gait. Using PCA, we could explain a total variance of 90%, 95%, and 99% with 30, 50, and 120 principal components, respectively. The first four principal components shown in Fig. 3 were multiplied with a score and added to the mean gait pose. The first and second principal components represented the flexion/extension of the shoulder joint and internal/external rotation of the foot, respectively. The third principal component represented the forward-directional angle of the lower body, resulting in a difference in the amplitude of leg movement. The fourth principal component represented hip and knee joint rotations. The detailed correlation coefficients between the first eight principal components and gait parameters are shown in Fig. 3.

### Intra-subject variance and variance ratio

**Figure 4:**
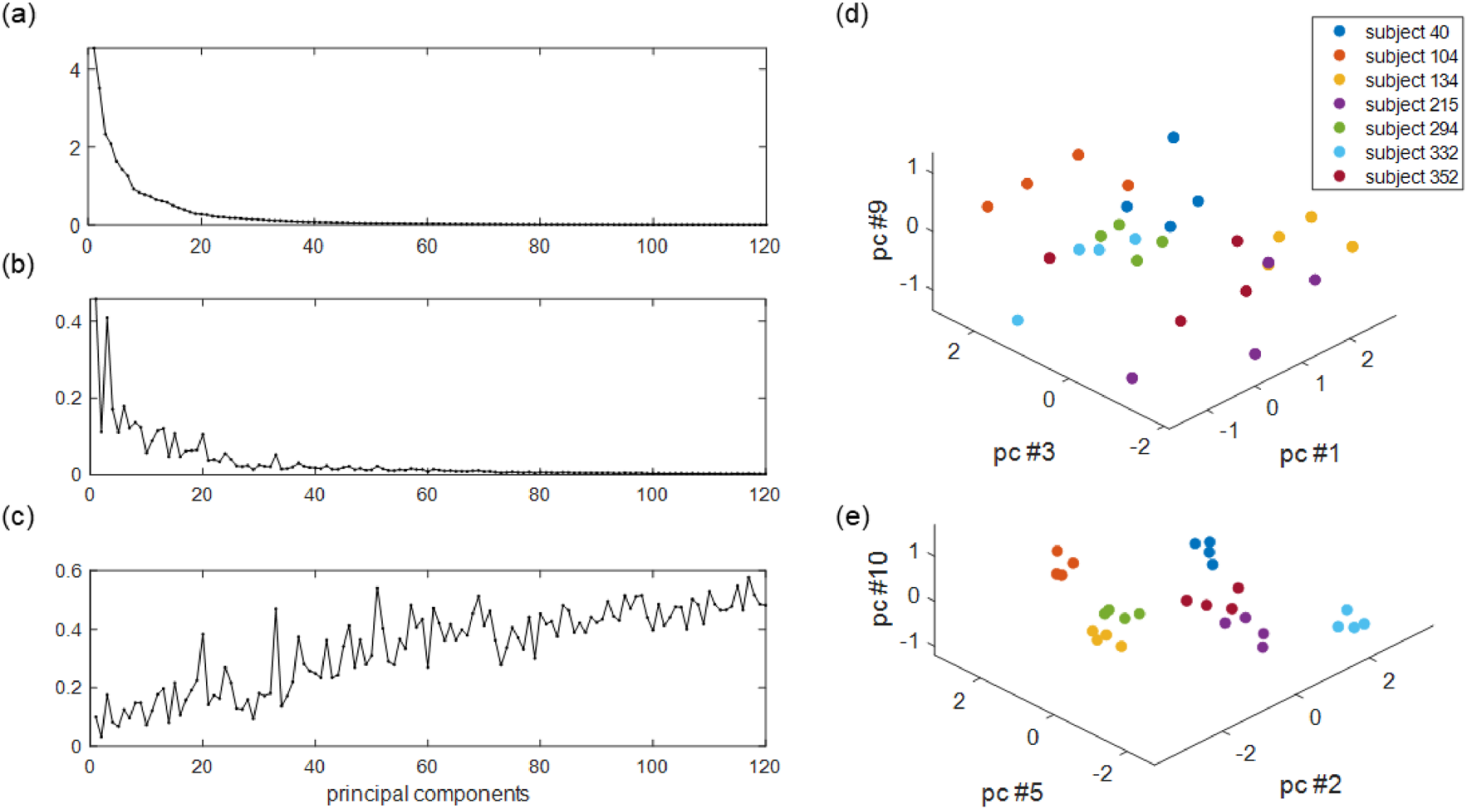
**(a–c)** Plot of variance properties of 120 principal components. **(a)** Plot of the explained variance. **(b)** Plot of the intra-subject variance. **(c)** Plot of the intra-subject variance to the explained variance ratio. **(d,e)** Data distribution with example set of principal components.

A gait feature vector whose components were the scores of the principal components were calculated for each gait vector. For each principal component, the intra-subject variance of the scores from the four gait vectors of each subject was calculated and averaged over 488 subjects. The variances calculated from the entire set of gait vectors are shown in Fig. 4a, and the average intra-subject variances of the first 120 principal components are shown in Fig. 4b. The variance from the entire set is the same as the explained variance of the principal component. Because principal components were numbered in the order of their explained variance, it decreased monotonically for the latter principal components. However, the average intra-subject variance did not decrease monotonically. This implies that the intra-subject variance of the principal component is not strictly proportional to its total variance. A feature with lower intra-subject variance would be more consistent for an individual when there are two features of gait with similar total variance. Likewise, if a feature shows a higher total variance than the other for the same intra-subject variance, the feature is more discriminative for different people. Therefore, we calculated the intra-subject variance to explained variance ratio to consider both intra-subject variance and total variance.

The intra-subject variance-to-explained variance ratios are shown in Fig. 4c. In Fig. 4d,e, we compared two sets of principal components. The first set with the first, third, and ninth principal components displayed relatively high intra-subject variance to explained variance ratios ranging from 0.101 to 0.176. The second set with the second, fifth, and tenth principal components had relatively low intra-subject variance to explained variance ratios ranging from 0.032 to 0.073. Figure 4d,e show the distribution of data in the feature space of the principal components in each set. The axes correspond to the scores of the principal components, and the color of each point represents individual subjects. The first set with higher intra-subject variance was used as the feature set in Fig. 4d, and the second set with lower intra-subject variance was used, as shown in Fig. 4e. We can observe in Fig. 4d,e that the data are more clearly separated for smaller intra-subject variance.

### Performance of individual verification

**Figure 5:**
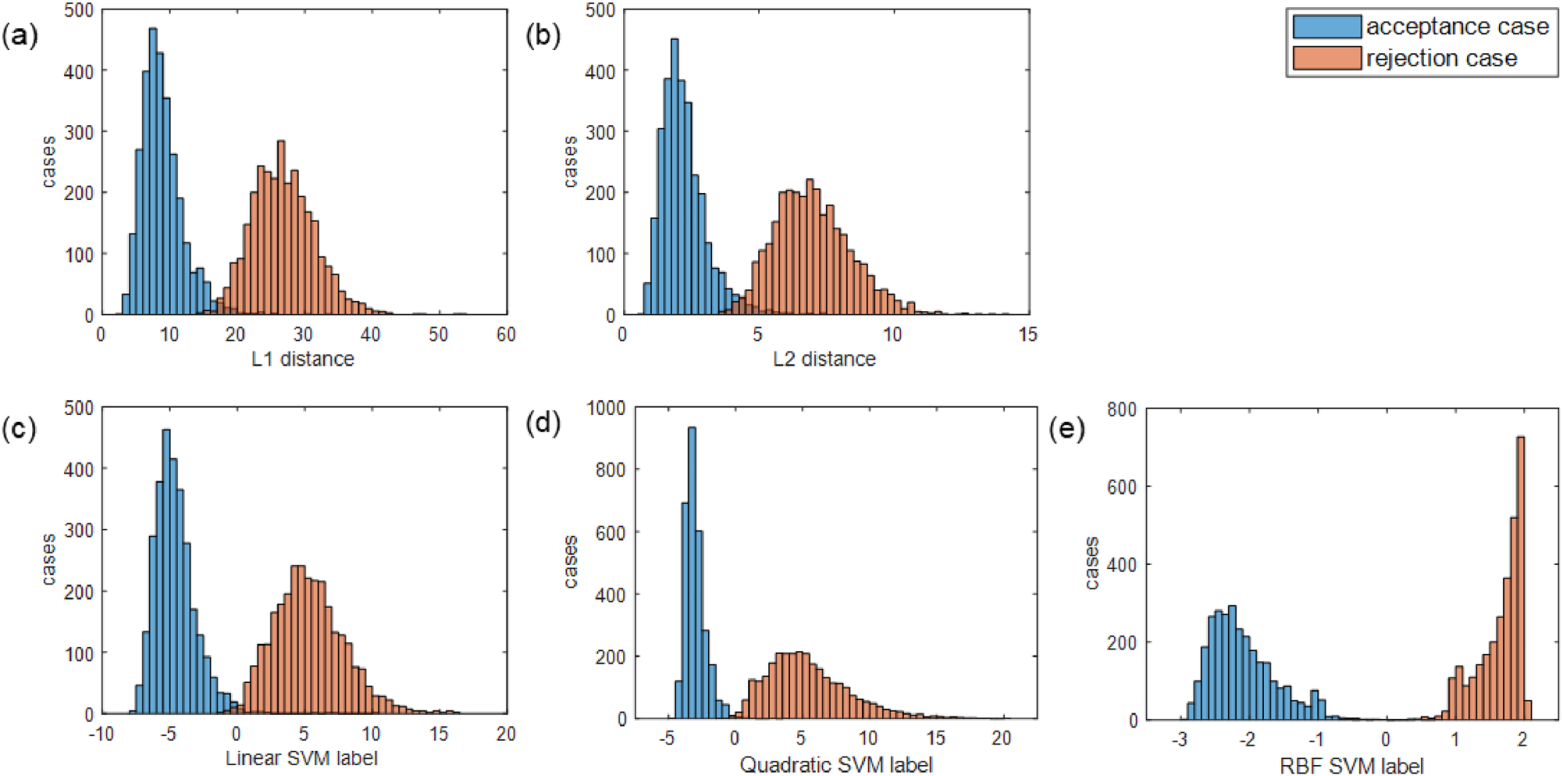
Distribution of classifier outputs, from acceptance case and rejection case pairs. The acceptance cases are displayed as blue histogram, while rejection cases are displayed as red histogram. Each of five figures corresponds to L1 norm calculation, L2 norm calculation, and SVMs with three kernels. The horizontal axis represents the output values from the classifiers.

The verification models with five different classification algorithms were tested for the first 30 principal components in the order of total variance. The distributions of the classifier outputs are shown as histograms for the acceptance and rejection cases in Fig. 5. In the first two figures illustrating L1 and L2 norms, the horizontal axis represents the L1 and L2 distances between the two gait feature vectors. Most of them ranged from 0 to 45 for L1 norms and from 0 to 12 for L2 norms. The average values at equal error rate (EER) were 18.4 for L1 norms and 4.6 for L2 norms. The horizontal axis in the figures illustrating support vector machine (SVM) classifier output denotes the distance from the separating hyperplane. The positive and negative values indicate that the classifier predicted that the two gait feature vectors were from the same person and from different persons, respectively. The blue and red histograms correspond to the distribution of the acceptance and rejection cases, respectively. The overlapping area of the two histograms implies that misclassified ambiguous cases exist. The histograms illustrating the SVM results had a smaller overlapping area than the histograms illustrating simple distance calculations.

**Figure 6:**
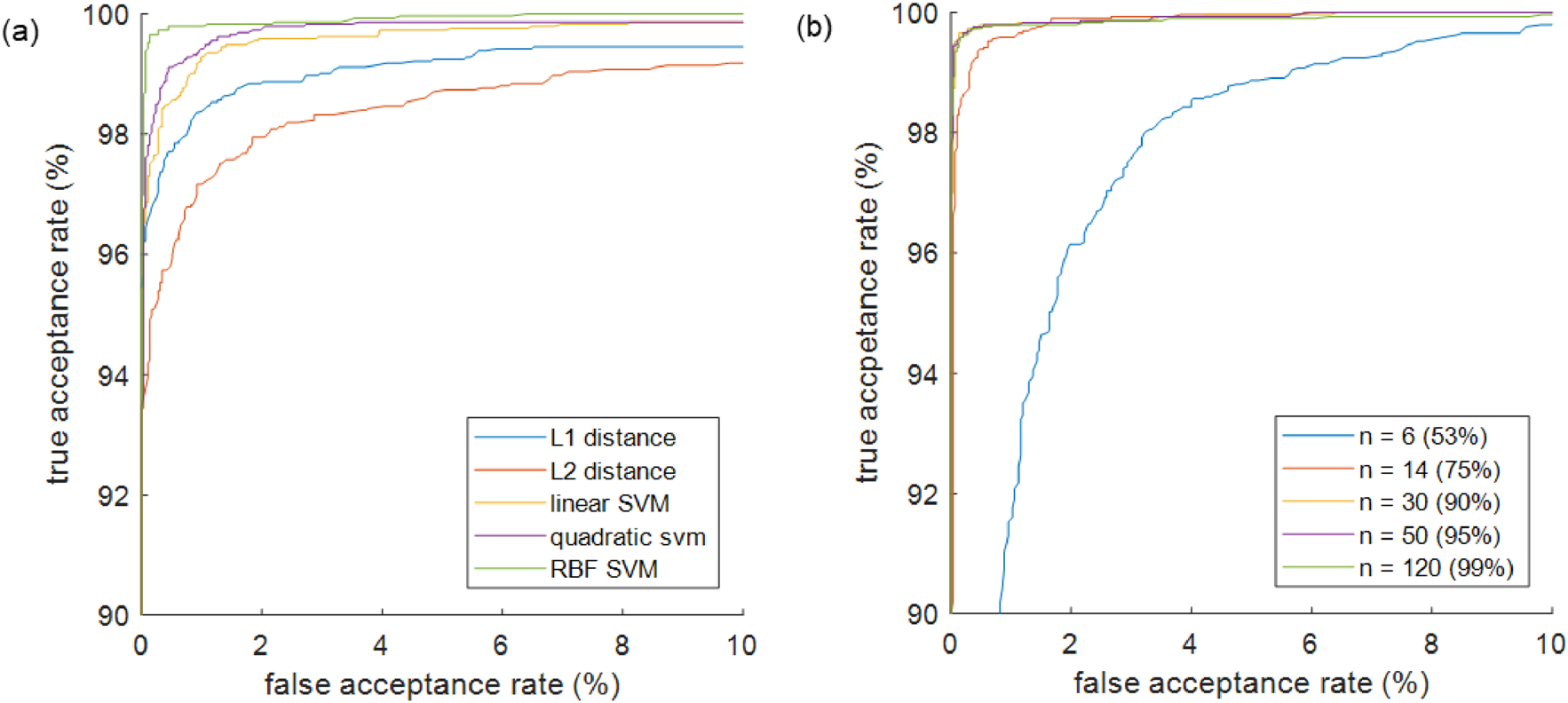
Plots of ROC curves for different classifier settings. **(a)** Comparison of five classification algorithms. **(b)** Comparison of gait feature vectors with different numbers of principal components.

According to the receiver operating characteristic (ROC) curve in Fig. 6, the SVM with the radial basis function (RBF) kernel displayed the best result. It could classify acceptance and rejection cases with 99.71% accuracy on the test set. In addition, the EER was just 0.27%. Therefore, in a population of randomly selected 300 people, a person will show a similar gait with only one of the other people on an average. Table 1 shows the classification accuracies and EERs of the SVM with RBF for different numbers of principal components: 6, 14, 30, 50, and 120. The accuracy improved when the number of principal components increased to 30. However, no significant change was observed in accuracy when the number of principal components was increased above 30.

**Table 1:** Classification accuracy and EER of SVM with the RBF kernel.

# PCs	Accuracy (%)	1—Accuracy (%)	EER (%)
6	97.37	2.63	2.77
14	99.32	0.68	0.55
30	99.71	0.29	0.27
50	99.69	0.31	0.27
120	99.69	0.31	0.34

## Discussion

The results showed that gait kinematics with segment orientation during walking could achieve an EER of 0.27% in the verification problem. Kaye^18^ defined special uniqueness as uniqueness conditioned by the given data. In our study, the given data were a sequence of gait kinematics from a person. EER is the probability of similar gait kinematics being found in another unrelated person within the population. Let us consider a population of 10 people and a sequence of gait kinematics data from one of them. The probability of an error in finding the owner of gait kinematics is at most 2.7% (0.27% × 10). Moreover, the sequence of gait kinematics can be assumed to have at least a 97.3% chance of being unique within the population. Conversely, general uniqueness means that every person in a given population possesses features with specific uniqueness^18^. The probability that all 10 individuals in the population will have unique gait kinematics is approximately 76.1% (97.3%^10^). The estimation of both special and general uniqueness depends on the population size. A much lower EER would be required to use the biometric for identification in a larger population.

The EER of the gait was not as low as the EER of the fingerprint, which was 0.022% in fingerprint verification competitions^30^. New biometric features are being developed and verified based on recent multimodal approaches. For example, lip motion during speaking achieved an EER of 8%^21^, and ratiometric finger impedance achieved an EER of 3.00%^24^. Although EERs depend on the size and quality of the dataset, the EER of the gait kinematics is comparable to the EERs of the new biometric features. The advantage of gait kinematics is that it can be measured even from a distance through video.

The gait vector in our study consisted only of body kinematics, without body segment lengths. Studies have been conducted on the uniqueness of somatotypes in populations^31,32^, but we focused on the movement pattern of an individual. Gait is created by complex interactions between the neural control of muscles and human body dynamics^33^. We focused on behavioral biometric information and investigated the uniqueness of human gait. Furthermore, information regarding the body segment orientation is more extensively used for studies on clinical gait analysis. Many previous studies selected joint rotation, flexion, and abduction as features rather than the relative coordinates of joints for the analysis of gait uniqueness^34^.

We used the PCA method to reduce the dimensions of the gait vector that had 4848 dimensions. Determining the statistical classification boundary for high-dimensional data requires large samples. Acquiring experimental gait data is expensive and laborious. Hence, effective data reduction is frequently used for statistical analysis of gait uniqueness^35^. PCA can extract linear components of the gait data in the order of the amount of information, variance. PCA demonstrates superior performance to nonlinear dimension reduction methods for certain datasets^36^.

The relationships between the traditional gait features and principal components extracted from the gait vectors are shown in the results. The first principal component with high intra-subject variance and total explained variance had a high correlation with upper-body movement. Therefore, upper-body movement has higher variance than other parts of the body while walking, even for the same person. The first principal component was also correlated to the walking speed. This supports the idea that walking speed is related to the flexion and extension of the upper body joints^37^. The subjects in our study walked at a self-selected speed. Therefore, walking speed influenced the high intra-subject variance of the first principal component. The second principal component, which is related to the rotation of the foot, showed the smallest intra-subject variance to the explained variance ratio providing statistical grounds for using ankle movements for person identification applications. Foot inversion/eversion and outward rotation of the ankle were used to specify the subject in a previous study using gait from videos for forensic investigation^38^.

To recognize individuals based on gait features, such features should be invariant for the same person^3^. To compare variations of each subject and variation among all the subjects at once, we calculated intra-subject variance to explained variance ratio for each principal component. A small intra-subject variance to explained variance ratio means that the score variance of the principal component is small for the same person, compared to the total score variance. The two example sets of the principal components in the results are features with low intra-subject variance (components 2, 5, and 10) and high intra-subject variance (components 1, 3, and 9). We observe in Fig. 4 that a set of robust features simplifies subject classification.

In many studies using PCA, principal components with a lower variance are regarded as less important features^39^. The PCA could reduce the dimension of gait vectors efficiently. The performance of the binary classifier increased when we used the principal components up to 30. The intra-subject variance to explained variance ratio generally increased for principal components greater than 30. We tried to change the order of the principal components from higher explained variance first to lower intra-subject variance to explained variance first. Using less than four principal components improved the classification accuracy of the SVM with the RBF kernel. The classification accuracy was improved from 69.45 to 78.93% for one principal component, 87.28% to 88.18% for two principal components, and 90.35% to 92.93% for the three principal components. However, it did not change significantly for four principal components.

Among binary classification algorithms, SVMs showed higher performance than L1 or L2 norms. While L1 and L2 norms classify data with one threshold, SVM can optimize its classification boundary using more parameters. In other words, SVM can determine approximate weightage for each principal component according to its effect on the classification performance. Among SVMs, the SVM with the RBF kernel achieved the highest classification accuracy. For our data, which are the differences between two gait feature vectors, we assumed the acceptance case to have a smaller magnitude. Therefore, the classification boundary between the acceptance and rejection cases had a shape that enclosed data corresponding to the acceptance case. This shape of the boundary was obtained using an SVM with the RBF kernel. For the other methods, we used an absolute value from each component of the gait feature vectors. The main reason that the SVM with RBF kernel achieved the best performance might be that there was no information loss due to using absolute values. The multilayer perceptron model from PyTorch was also tested using the same binary classification problem. Using a multilayer perceptron as a classification algorithm resulted in the highest classification accuracy of 99.35%. Adding more layers to the classification model could not further improve the classification accuracy.

Our study quantified the uniqueness of gait using the well-established PCA and SVM methods. The gait feature vector was obtained by applying PCA, which is a linear dimensionality reduction method, to the gait vectors. Nonlinear dimensionality reduction methods such as autoencoders can perform better depending on the characteristics of the data^40^. In addition, state-of-the-art deep learning methods, such as convolutional neural networks, can improve the classification accuracy even without the dimensionality reduction of the input data^14–16^. Thus, the uniqueness of the gait should be further validated using state-of-the-art methods. Another limitation is that the uniqueness of gait is quantified with only a standard walking motion in this study. Thus, the results of this study should be used carefully for person identification. The uniqueness of motions other than gait can be different from the results of this study. The number of subjects in our study was not as large as that in the video-based motion datasets^41^. Optoelectronic motion capture systems provide a state-of-the-art accuracy in measuring three-dimensional gait motions^27,28^; therefore, the measurement errors can be minimized to understand the uniqueness of gait.

The EER of gait kinematics was quantified from the three-dimensional gait data obtained in a controlled clinical environment using state-of-the-art motion capture system that could provide high accuracy for joint positions. The EER of gait kinematics would increase if the three-dimensional gait kinematics were estimated using a sequence of videos taken from a single viewpoint, such as from a closed-circuit camera. However, the rapid development of computer vision techniques, such as body keypoint detection^9^ and three-dimensional pose reconstruction^10–12^ will be able to provide accurate three-dimensional gait kinematics with a single camera in the near future. In our study, the gait data of each subject were obtained during a single visit to the motion capture laboratory. Several movements of a subject were captured in a short period of time. Changes in gait kinematics with respect to weight, injury, and aging need to be studied further^4^. To the best of our knowledge, this is the first study to assess the potential of full-body kinematics during walking as a unique characteristic of an individual.
